# Fermentation of glycerol by *Anaerobium acetethylicum* and its potential use in biofuel production

**DOI:** 10.1111/1751-7915.12484

**Published:** 2016-12-22

**Authors:** Yogita Patil, Madan Junghare, Nicolai Müller

**Affiliations:** ^1^ Department of Biology, Microbial EcologyUniversity of KonstanzKonstanzD‐78457Germany; ^2^ Konstanz Research School of Chemical BiologyUniversity of KonstanzKonstanzD‐78457Germany

## Abstract

Growth of biodiesel industries resulted in increased coproduction of crude glycerol which is therefore becoming a waste product instead of a valuable ‘coproduct’. Glycerol can be used for the production of valuable chemicals, e.g. biofuels, to reduce glycerol waste disposal. In this study, a novel bacterial strain is described which converts glycerol mainly to ethanol and hydrogen with very little amounts of acetate, formate and 1,2‐propanediol as coproducts. The bacterium offers certain advantages over previously studied glycerol‐fermenting microorganisms. *Anaerobium acetethylicum* during growth with glycerol produces very little side products and grows in the presence of maximum glycerol concentrations up to 1500 mM and in the complete absence of complex organic supplements such as yeast extract or tryptone. The highest observed growth rate of 0.116 h^−1^ is similar to that of other glycerol degraders, and the maximum concentration of ethanol that can be tolerated was found to be about 60 mM (2.8 g l^−1^) and further growth was likely inhibited due to ethanol toxicity. Proteome analysis as well as enzyme assays performed in cell‐free extracts demonstrated that glycerol is degraded via glyceraldehyde‐3‐phosphate, which is further metabolized through the lower part of glycolysis leading to formation of mainly ethanol and hydrogen. In conclusion, fermentation of glycerol to ethanol and hydrogen by this bacterium represents a remarkable option to add value to the biodiesel industries by utilization of surplus glycerol.

## Introduction

Fossil fuels are the main source of energy being used worldwide and cover about 80% of the global energy demand (Sarma *et al*., 2012). Fossil fuels are limited, non‐renewable and associated with many problems such as global warming, ecosystem imbalance and health hazards (da Silva *et al*., 2009). Therefore, there is a huge demand for alternative energy sources that are renewable, eco‐friendly and sustainable to replace the conventional fossil fuels. Moreover, Campbell and Laherrere (1998) predicted that petroleum reserves will be completely depleted by 2050 (Nwachukwu *et al*., 2012). This concern has highlighted the future need for the use of biofuels such as ethanol, biodiesel, butanol, hydrogen or electricity produced from renewable plant biomass as one of the promising alternatives over fossil fuels (Elmekawy *et al*., 2013; Speers *et al*., 2014). Therefore, in recent years there has been a significant increase in the production and use of biofuels worldwide, such as biodiesel and bioethanol.

In the last decade, the European Union (EU) was the principal biodiesel producer which contributed about 82% of global biodiesel production (Demirbas and Balat, 2006). According to the European Biodiesel Board (EBB, 2006), the estimated production of biodiesel in 2005 was about 3.2 million tons with a production capacity of 6 million tons (da Silva *et al*., 2009), which has now increased to about 10.4 million tons in 2013 with a production capacity of 23 million tons. Germany is currently the largest producer and consumer of biodiesel in the EU, producing more than 2.5 million tons in 2013 (EBB, 2013; http://www.ebb-eu.org/stats.php). The top five global producers of biodiesel are Argentina, Brazil, France, Germany and the United States of America (Sarma *et al*., 2012).

Glycerol (1,2,3‐propanetriol) is a simple trivalent alcohol that results from the natural degradation of the glyceride component of plant cell wall phospholipids or reserve lipids of plant seeds (Roger *et al*., 1992; Nwachukwu *et al*., 2013). It is produced in major amounts during transesterification of vegetable oils and animal fats (Solomon *et al*., 1995; Barbirato *et al*., 1997a,b, 1998; Colin *et al*., 2001) and has wide applications in different industries such as food and drinks, toothpaste, cosmetics, toiletries, plastics, tobacco, pulp and paper, paint, leather and textile, pharmaceuticals and automotive (Choi, 2008; Nicol *et al*., 2012; Rossi *et al*., 2012). The economic value of industrial glycerol has decreased due to the surplus crude glycerol generated during biodiesel production, and it cannot be utilized directly in any industrial applications due to the presence of impurities. Furthermore, it cannot be directly released into the environment without treatment as the cost of such treatment is not economical (Nwachukwu *et al*., 2013). Recently, fermentative conversion of crude glycerol into valuable products such as, e.g., bioethanol has gained interest for the development of biodiesel‐producing industries, and also for replacing conventional carbohydrate sugars used in industrial microbial fermentation processes to convert it into a broad range of value‐added organic products such as bioethanol (Dharmadi *et al*., 2006; Rossi *et al*., 2012).

Bioethanol is considered as an alternative to fossil fuels, as it is a renewable, bio‐based resource, and provides the potential to reduce particulate emissions (Hansen *et al*., 2005). Several microorganisms produce ethanol as a natural fermentation end‐product, sometimes even in a homo‐ethanologenic type of fermentation (Otero *et al*., 2007). Bioethanol is one of the fermentation products that can be generated from glycerol via anaerobic fermentation, which is more economical than the use of corn or lignocellulosic biomass for bioethanol production (Choi, 2008). Moreover, the cost of ethanol produced from glycerol is about 40% lower than when it is produced from corn (Yazdani and Gonzalez, 2007).

Fermentation of glycerol most often leads to 1,3‐propanediol as reduced end‐product (Homann *et al*., 1990). *Escherichia coli* was shown to ferment glycerol anaerobically to ethanol, hydrogen and formate, thus providing a bioagent to produce value‐added biofuel from glycerol (Dharmadi *et al*., 2006; Trchounian and Trchounian, 2015). Other microorganisms are able to perform similar fermentations of glycerol, especially several members of the genus *Clostridium* (Biebl, 2001). Also mixtures of microorganisms, e.g. buffalo slurry, were used to optimize hydrogen production from glycerol (Marone *et al*., 2015). The main problems with glycerol‐fermenting bacteria are the accumulation of undesired by‐products such as 2,3‐butanediol or butyric acid, and the low tolerance of these strains towards solvents, i.e. glycerol and ethanol. The latter two dissolve cellular membranes at higher concentrations and are therefore lethal for any kind of microorganism. However, yeasts can tolerate ethanol concentrations up to about 120 g l^−1^ (15% v/v; Lam *et al*., 2014), which is similar to some bacteria, e.g. *Zymomonas* sp. (Swings and De Ley, 1977).

Recently, an anaerobic bacterium representing the new genus *Anaerobium* within the order *Clostridiales* was enriched and isolated from sludge samples obtained from a biogas reactor at Odendorf, Germany. *Anaerobium acetethylicum* strain GluBS11^T^ was originally described for gluconate fermentation, but it grows also with glycerol under strictly anoxic conditions (Patil *et al*., 2015). Unlike many other members of the order *Clostridiales*, fermentation of glycerol by *A. acetethylicum* mainly produces ethanol and hydrogen and does not coproduce undesired by‐products such as butyrate, 1,3‐propanediol or 2,3‐butanediol under any growth condition (Patil *et al*., 2015). In this study, we describe the optimum conditions for glycerol fermentation to ethanol and hydrogen by *A. acetethylicum* using pure glycerol at different concentrations and elucidate the biochemical reactions involved in anaerobic glycerol fermentation based on proteomics and *in vitro* enzyme assays. Based on our findings, we propose a glycerol fermentation pathway that mainly leads to ethanol and hydrogen and does not involve the formation of 1, 3‐propanediol or 2,3‐butanediol. Application of *A. acetethylicum* as a potential future candidate for bioethanol and biohydrogen production from glycerol is discussed in the context of the proposed pathway.

## Results

### Anaerobic glycerol fermentation by *A. acetethylicum*

**Figure 1:**
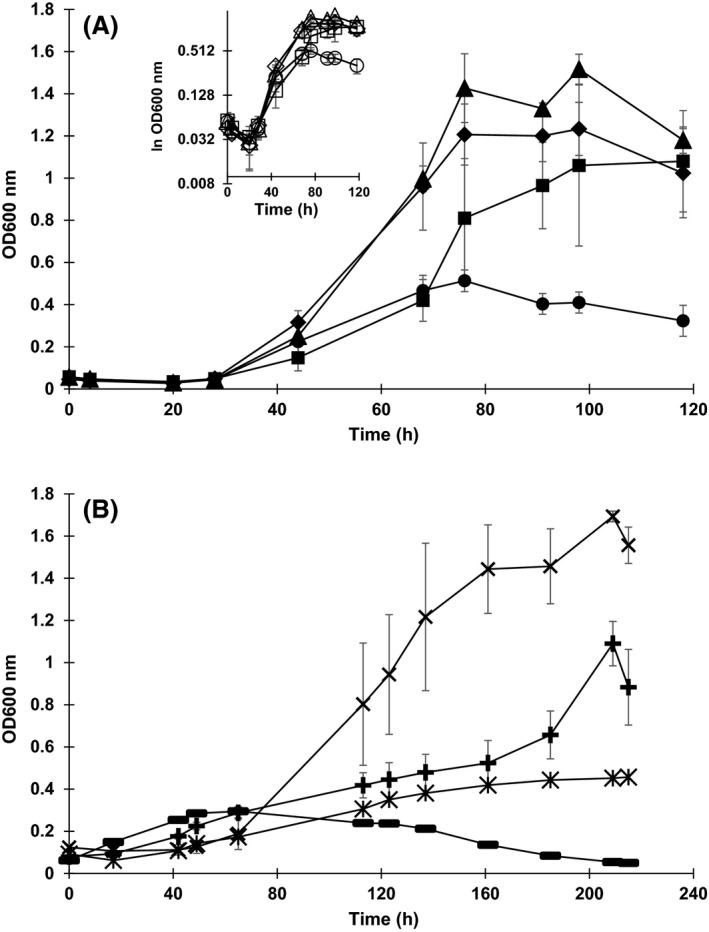
Growth of *Anaerobium acetethylicum* at different initial concentrations of glycerol incubated at 30°C for 118 h. Shown are mean values of triplicates ± standard deviations, except for 1500 mM of glycerol (*n* = 2). A. ● circles: 10 mM of glycerol, ♦ diamonds: 50 mM of glycerol, ▲ triangles: 100 mM of glycerol, ■ squares: 200 mM of glycerol. A inset. Half‐logarithmic plot of the data presented in (A). Same symbols as in (A), but open symbols. B. X xes: 500 mM of glycerol, + crosses: 1000 mM of glycerol, * asterisks: 1500 mM of replicate A, − minuses: 1500 mM of replicate B.

**Figure 2:**
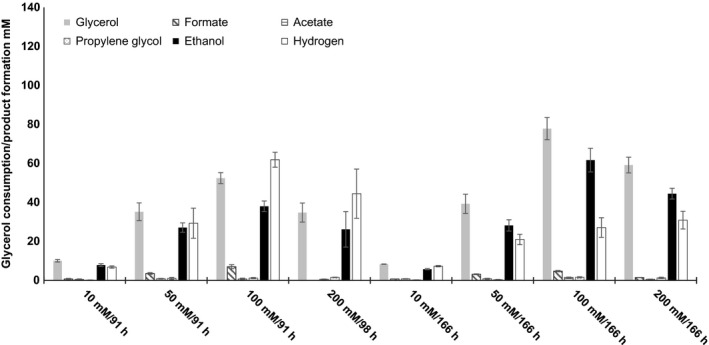
Glycerol consumption and product formation in cultures with varying initial glycerol concentrations. Products and substrate were analysed after 91 h, 98 h or 166 h as indicated. Headspace‐to‐culture volume ratio 1.5. Shown are mean values of triplicates ± standard deviations, except for 200 mM/98 h (*n* = 2). Order of bars in the respective category: first glycerol, second formate, third acetate, fourth propylene glycol, fifth ethanol, sixth hydrogen.

In strictly anaerobic growth experiments with *A. acetethylicum,* glycerol is fermented mainly to ethanol, hydrogen and small amounts of acetate, formate and propylene glycol (1,2‐propanediol). In cultures with different initial concentrations of glycerol (10, 50, 100 and 200 mM added to cultures, actual concentrations 10.8, 48.7, 97.4 and 189.6 mM, respectively), the strain exhibited different lag phase periods which increased with increasing glycerol concentrations (Fig. 1A and B). Maximal growth rates (μ_max_) observed in the exponential phase were 0.101, 0.116, 0.107 and 0.070 h^−1^ at 10, 50, 100 and 200 mM of glycerol respectively (Fig. 1A). Growth was exponential within 28 and 44 h as shown in the half‐logarithmic plot (Fig. 1A inset). After 44 h, growth was not exponential any more (Fig. 1A). At all tested glycerol concentrations, the strain consumed glycerol within 91–98 h of incubation and produced ethanol and hydrogen as major fermentation products, whereas the concentrations of formate, acetate and propylene glycol were comparatively low (Table 1). The maximum ethanol concentration produced after 91–98 h of incubation was about 38 mM when the cells were cultivated with 100 mM of initial glycerol (Fig. 2). In another growth experiment, final concentrations were measured after 166 h, i.e. when the cells were in late stationary to decline phase, and the maximum average ethanol concentration observed was 62 mM at 100 mM of initial glycerol concentration (Fig. 2, growth curve not shown).

**Table 1:** Stoichiometry of glycerol fermentation and product formation by *Anaerobium acetethylicum* at different initial glycerol concentrations after 98 h. Shown are mean values of *n* = 3 except for the growth experiment with 189.6 mM of glycerol (*n* = 2)

Glycerol					Fermentation products (mM)					Yield	
Initial (mM)	Cell dry mass formed (mg l^−1^)	Consumption (mM)	Assimilation (mM)^a^	Dissimilation (mM)	Ethanol	H_2_	Formate	Acetate	Propylene glycol	Electron recovery (%)	Growth yield (g dry mass mol^−1^ substrate)
10.8	114	10.1	1.3	8.8	7.9	6.9	0.7	0.5	0.1	93.8	11.3
48.7	304	35.2	3.6	31.6	27.1	29.3	3.6	0.8	1	93.4	8.6
97.4	327	52.4	3.9	48.5	38	61.9	7	0.7	1.2	91.1	6.2
189.6^b^	217^b^	34.8^b^	2.6^b^	32.2^b^	26.2^b^	44.5^b^	0^b^	0.6^b^	1.6^b^	96.9^b^	6.2^b^
506.1	284.9	71.3	3.4	67.9	57.9	69	0	0.5	2	91.4	4

**a.** Glycerol assimilated was calculated assuming an OD_600_ to dry mass correlation of 250 mg l^−1^ = OD_600_ 1 and according to the following assimilation equation: 17 C_3_H_8_O_3_ + 5 CO_2_ → 14 < C_4_H_7_O_3_ > + 19 H_2_O.

**b.** Duplicate measurements, one culture with 189.6 mM of glycerol did not grow, all other measurements done in triplicate.

At the end of the growth experiments, cultures were analysed for glycerol consumption and assimilation (maximum growth at late exponential phase) and dissimilation to different fermentation products. The electron recoveries obtained from the fermentation of 10, 50, 100, 200 and 500 mM of glycerol were found to be in a range of 91.1–96.9%, indicating that no major fermentation products beyond ethanol, hydrogen, and small amounts of formate, acetate and propylene glycol were formed (Table 1). However, at glycerol concentrations higher than 500 mM and when cultures were in late stationary to decline phase, the electron recoveries were lower than 90% (data not shown), indicating that part of the products or the amount of assimilated glycerol was not quantified correctly. At 10 mM of initial glycerol, the cells converted 8.8 mM of glycerol to 7.9 mM of ethanol and 6.9 mM of hydrogen; i.e., the glycerol‐to‐ethanol ratio was found to be about 1: 0.9 (Table 1). Growth yields decreased with increasing substrate concentrations and were 11.3 g dry mass mol^−1^ substrate at 10 mM of initial glycerol and 4 g dry mass mol^−1^ substrate at 500 mM of initial glycerol (Table 1).

Another independent batch fermentation experiment with 100 mM of glycerol carried out in triplicates showed that the average pH value changed within 166 h from initially 7.2 to 6.3, probably due to the accumulation of CO_2_ as no significant amounts of organic acids could be identified.

Although the fermentation of glycerol by *A. acetethylicum* yielded mainly ethanol and hydrogen and small amounts of acetate, formate and propylene glycol, another yet unidentified and unquantified compound was observed. This compound had a retention time of 21.1 min under our separation conditions and did not correspond to any of numerous compounds tested that could possibly arise as side products of glycerol fermentation, e.g. 1,3‐propanediol, 3‐hydroxypropionaldehyde synthesized from acrolein, 1‐butanol, butyrate, succinate, 1,3‐butanediol, acetaldehyde, 2‐oxopropanal, 1‐propanol and 2‐propanol.

### Growth of *A. acetethylicum* at elevated glycerol concentrations: solvent toxicity

**Figure 3:**
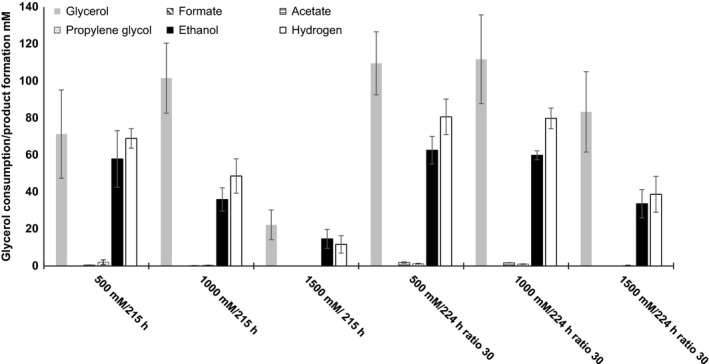
Glycerol consumption and product formation in cultures with varying initial glycerol concentrations. Products and substrate were analysed after 215 h or 224 h as indicated. Headspace‐to‐culture volume ratio 1.5 or 30 where indicated. Shown are mean values of triplicates ± standard deviations, except for 1500 mM/215 h (*n* = 2). Order of bars in the respective category: first glycerol, second formate, third acetate, fourth propylene glycol, fifth ethanol, sixth hydrogen.

In order to investigate the maximum tolerable glycerol concentration, growth experiments with varying initial glycerol concentrations ranging from 500 to 3000 mM were performed. The strain showed longer lag phases when precultures grown at lower glycerol concentrations were inoculated into medium containing higher initial glycerol concentrations. Therefore, a culture of *A. acetethylicum* was slowly adapted to higher concentrations by subsequently transferring inocula from stationary‐phase batch cultures with 10 mM of glycerol to 200 mM, to 500 mM, to 1000 mM and finally to 1500 mM of glycerol. Then, the growth experiments were repeated (Fig. 1B). At higher glycerol concentrations, growth was measurable at 500, 1000 and 1500 mM of glycerol. A maximum of 1500 mM of glycerol corresponding to 138 g l^−1^ was tolerated and growth was observed in two of three replicates with maximum optical densities at 600 nm of 0.46 and 0.3 at 1500 mM of initial glycerol concentration (Fig. 1B). Growth was completely inhibited at 2000 and 3000 mM of initial glycerol concentrations (data not shown). The growth tests were repeated and experimental conditions improved by the following modifications: cultivation was done in 600 ml infusion bottles with 20 ml of culture volume to increase the headspace‐to‐culture volume ratio to 30 thus allowing accumulation of higher amounts of hydrogen and subsequently ethanol, which is coproduced with hydrogen in an approximately 1:1 ratio. The maximally accumulated average ethanol concentration was 63 mM (2.9 g l^−1^) after 224 h of incubation when cultures were grown with 500 mM of initial glycerol in infusion bottles with a headspace‐to‐culture volume ratio of 30 (Fig. 3). Growth and glycerol degradation did not proceed further when the culture reached this ethanol concentration. In cultures with 500, 1000 and 1500 mM of initial glycerol concentrations with a headspace‐to‐culture volume ratio of 1.5, ethanol and hydrogen yields were generally slightly lower compared with cultures with the same initial glycerol concentrations and a headspace‐to‐culture volume ratio of 30 (Fig. 3).

### Identification of genes encoding putative enzymes involved in glycerol fermentation by *A. acetethylicum*

**Figure 4:**
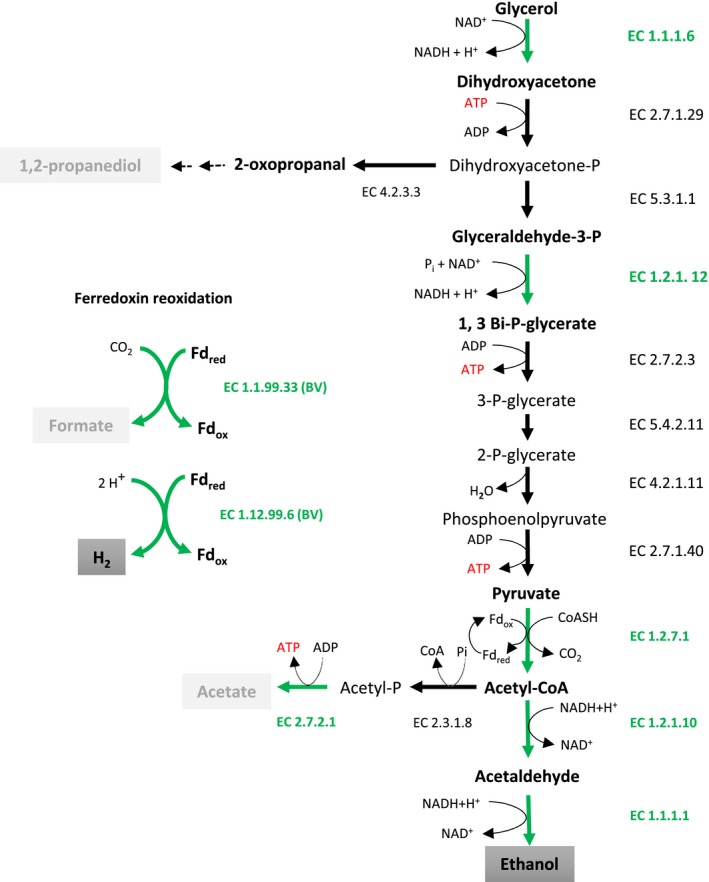
Anaerobic fermentative metabolism of glycerol by *Anaerobium acetethylicum* showing formation of the ethanol, acetate, formate and hydrogen. Green thick arrows indicate the activity of the respective enzyme confirmed by proteome analysis and enzyme assays with cell extract glycerol‐grown cells. Bold black arrows represent enzymes confirmed by total proteome analysis of glycerol‐grown cells and dashed arrows show putative steps for 1, 2‐propanediol production. The genes encoding (locus tag) for the respective enzymes involved in the glycerol metabolism are shown: glycerol dehydrogenase (EC 1.1.1.6; Ga0116910_101526 and 101551); dihydroxyacetone kinase (EC 2.7.1.29; Ga0116910_101527); triosephosphate isomerase (EC 5.3.1.1; Ga0116910_10001 and 102914); glyceraldehyde 3‐phosphate dehydrogenase (EC 1.2.1.12; Ga0116910_1001391); phosphoglycerate kinase (EC 2.7.2.3; Ga0116910_1001391); phosphoglycerate mutase (EC 5.4.2.11; Ga0116910_1001389 and 103027); enolase (EC 4.2.1.11; Ga0116910_1001503); pyruvate kinase (EC 2.7.1.40; Ga0116910_1004153); pyruvate‐ferredoxin oxidoreductase (EC 1.2.7.1; Ga0116910_103224); CoA‐dependent acetaldehyde dehydrogenase (EC 1.2.1.10; Ga0116910_1004188); alcohol dehydrogenase (EC 1.1.1.1; Ga0116910_101528 and 101313); phosphoacetyl transferase (EC 2.3.1.8; Ga0116910_1001587); acetate kinase (EC 2.7.2.1; Ga0116910_1001586); hydrogenase (EC 1.12.99.6; Ga0116910_100543); formate dehydrogenase (EC 1.1.99.33); and methylglyoxal synthase (EC 4.2.3.3; Ga0116910_1001113). BV – benzyl viologen and Fd – ferredoxin.

Whole‐proteome analysis of cell‐free extracts of glycerol‐grown cells of *A. acetethylicum* revealed that the genes coding for all putative enzymes involved in the proposed pathway shown in Fig. 4 were expressed during growth with glycerol. Glycerol is a small molecule that can diffuse slowly across the bacterial cell membrane. Facilitated diffusion is the least common type of energy‐independent transport systems found in bacteria, e.g. the glycerol uniporter in *E. coli* (Sweet *et al*., 1990; Truninger and Boos, 1993). Although we could not detect the presence of a glycerol uptake facilitator protein (GlpF) in the total proteome analysis yet, we were able to identify the corresponding gene locus tag (Ga0116910_10171) predicted to code for the GlpF protein in the genome of *A. acetethylicum*. According to the proposed glycerol fermentation pathway in *A. acetethylicum* (Fig. 4), after uptake (GlpF), glycerol is first converted to dihydroxyacetone (DHA) by glycerol dehydrogenase (GldA), which is subsequently phosphorylated to DHA‐phosphate (DHAP) by dihydroxyacetone kinase (DhaK). Proteome analysis identified two putative genes that encode GldA (locus tag Ga011691_101526 and 101551; in the following section, the prefix ‘Ga011691’ is omitted from the locus tag) and Dhak (101527; Table 2). DHAP is either partially metabolized to 1,2‐propanediol via 2‐oxopropanal, which involves a putative methylglyoxal synthase (Mgs; 1001113) or metabolized further by enzymes of the glycolytic pathway via acetyl‐CoA as shown in Fig. 4. As expected, candidate genes coding for the putative enzymes triosephosphate isomerase (10001 and 101914), glyceraldehyde 3‐phosphate dehydrogenase (1001392), phosphoglycerate kinase (1001391), phosphoglycerate mutase (1001389 and 103027), enolase (1001503), pyruvate kinase (1004153) and ferredoxin‐dependent putative pyruvate:ferredoxin oxidoreductase (103224) were expressed and identified in the proteome of glycerol‐grown cells of *A. acetethylicum* (Table 2). However, pyruvate kinase (1004153) had a sequence coverage of only 12.2% with three identified peptides (Table 2).

**Table 2:** Proteins that are likely to be involved in the proposed glycerol fermentation pathway identified by total proteomics (Orbitrap LC‐MS analysis) from cell‐free extract of *Anaerobium acetethylicum* cells grown with glycerol

Gene loci^a^	IMG predicted function^b^	Coverage^c^ (%)	Peptides^d^	Score^e^	Mass (KDa) f
101526	Glycerol dehydrogenase	55.15	12	8982	40.0
101551	Glycerol dehydrogenase	5.56	2	1894	40.5
101527	Dihydroxyacetone kinase	58.83	24	4911	62.2
1001390	Triosephosphate isomerase	89.92	11	4187	26.6
102914	Triosephosphate isomerase	67.56	12	1509	29.7
1001392	Glyceraldehyde 3‐phosphate dehydrogenase	70.92	15	3876	35.4
1001391	Phosphoglycerate kinase	78.34	20	5899	42.0
1001389	2,3‐bisphosphoglycerate‐independent phosphoglycerate mutase	31.91	11	861	56.6
103027	2,3‐bisphosphoglycerate‐independent phosphoglycerate mutase	14.68	4	176	44.5
1001503	Enolase	22.88	6	305	47.6
1004153	Pyruvate kinase	12.22	3	59	63.6
103224	Pyruvate‐ferredoxin/flavodoxin oxidoreductase	55.21	38	4261	127.9
1004188	Acetaldehyde dehydrogenase/alcohol dehydrogenase	83.01	58	22648	95.2
101528	Alcohol dehydrogenase	71.05	17	3608	39.8
101313	NAD(P)‐dependent dehydrogenase, short‐chain alcohol dehydrogenase family	10.63	2	158	27.4
1001587	Phosphate acetyltransferase	27.49	5	165	35.1
1001586	Acetate kinase	39.04	9	411	43.0
100543	Iron‐only hydrogenase large subunit, C‐terminal domain	12.24	5	88	64.9
1001113	Methylglyoxal synthase	52.53	6	258	17.6
1004109	Formate C‐acetyltransferase	38.53	13	562	75.7

**a.** Integrated microbial genomes (IMG; Markowitz *et al*., 2009) gene locus tag (Ga0116910_).

**b.** Protein identification and function derived from IMG annotation.

**c.** Sequence coverage represents the extent of peptides obtained during MS‐MS identification of the respective protein.

**d.** Number of peptides detected during MS‐MS identification.

**e.** Mascot search score; and f, peptide mass calculated by MS‐MS identification.

**f**. peptide mass calculated by MS‐MS identification.

Acetyl‐CoA is partly utilized for assimilation or it is further metabolized either to acetate or ethanol. Conversion of acetyl‐CoA to ethanol involves two enzyme reactions catalysed by acetaldehyde dehydrogenase and alcohol dehydrogenase respectively (Fig. 4). Proteome analysis identified the putative candidates for both enzymes, acetaldehyde dehydrogenase (1004188) and alcohol dehydrogenase (101528 and 101313; Table 2). Similarly, conversion of acetyl‐CoA to acetate involves two enzyme steps catalysed by phosphate transacetylase and acetate kinase (Fig. 4), which were both expressed and identified (phosphate transacetylase (1001587) and acetate kinase (1001586; Table 2). During oxidative decarboxylation of pyruvate to acetyl‐CoA by the ferredoxin‐dependent pyruvate:ferredoxin oxidoreductase, carbon dioxide (CO_2_) and reduced ferredoxin are generated. The released CO_2_ can be reduced to formate coupled to generation of oxidized ferredoxin by formate dehydrogenase (Fig. 4). Although glycerol fermentation by *A. acetethylicum* produced comparatively low amounts of formate (Table 1), proteome analysis did not confirm expression of candidate genes coding for putative formate dehydrogenases. Moreover, the draft genome sequence of *A. acetethylicum* does not contain candidate genes for formate dehydrogenases. Yet, a formate C‐acetyltransferase (pyruvate: formate lyase) was identified in the proteome (Table 2). Likewise, ferredoxin could also be reoxidized coupled to hydrogen production as show in Fig. 4, but hydrogenase could not be reliably detected in the proteome. The only potential candidate was an iron‐only hydrogenase (100543) with a sequence coverage of 12.2% and a score of 88.4 (Table 2). The coverage of most proteins identified were in the range of 31.9–89.9% (Table 2) and some identified proteins had coverages lower than 30% which makes it doubtful whether these were present in glycerol‐grown cells.

### Glycerol fermentation by *A. acetethylicum*:* in vitro* enzyme activity measurements

Key enzymes of a hypothetical pathway of glycerol fermentation to ethanol and hydrogen were assayed in *in vitro* enzyme assays with cell‐free extracts of glycerol‐ or glucose‐grown cells (the latter as a control). In the first step, glycerol dehydrogenase (GldA) oxidizes glycerol to dihydroxyacetone (DHA) with NAD^+^ as electron acceptor. Glycerol‐grown cells showed high specific activity for GldA (100 mU mg^−1^ of protein) with glycerol and NAD^+^. DHA is most likely phosphorylated to dihydroxyacetone phosphate (DHAP) by the activity of the enzymes dihydroxyacetone kinase and triose phosphate isomerase (Fig. 4). *In vitro* enzyme activities for methylglyoxal synthase, dihydroxyacetone kinase and triosephosphate isomerase were not assayed, but their involvement in the predicted glycerol fermentation pathway was evidenced by total proteome analysis (Table 2). Glyceraldehyde‐3‐phosphate was oxidized and phosphorylated to 1,3‐bisphosphoglycerate in cell‐free extract in the presence of NAD^+^ by glyceraldehyde‐3‐phosphate dehydrogenase, which showed an activity of 282 mU mg^−1^. 1,3‐bisphosphoglycerate is converted to pyruvate most likely by glycolytic enzymes which were not assayed but identified by whole‐proteome analysis (Table 2). Pyruvate is oxidized and decarboxylated to acetyl‐coenzyme A with ferredoxin by a putative pyruvate:ferredoxin oxidoreductase that could be measured using benzyl viologen (457 mU mg^−1^) as an artificial electron acceptor and was also confirmed by measuring the reduction of native oxidized ferredoxin (17 mU mg^−1^) prepared from *Clostridium pasteurianum* (Table 3).

**Table 3:** Measurement of key enzyme activities in the proposed pathway of glycerol fermentation by *A. acetethylicum* in cell‐free extract of cells grown with glycerol

Enzymes	Activity (mU mg^−1^ of protein)
Acetaldehyde dehydrogenase	37 ± 4.6
Alcohol dehydrogenase	513 ± 128
Glyceraldehyde 3‐P dehydrogenase	282 ± 47
Glycerol dehydrogenase	100 ± 28
Pyruvate‐ferredoxin oxidoreductase	457 ± 11 (BV) 17 ± 4 (Fd)
Formate dehydrogenase	1.6 ± 0.11
Hydrogenase	3070 ± 779 (BV)
Acetate kinase	26 ± 2.44

Enzyme activity measured with BV – benzyl viologen and Fd – ferredoxin.

Acetyl‐CoA can be reduced to ethanol with NADH via acetaldehyde by acetaldehyde dehydrogenase and alcohol dehydrogenase respectively (Fig. 4). Enzyme assays with glycerol‐grown cell‐free extract showed activity for acetaldehyde dehydrogenase (37 mU mg^−1^) and alcohol dehydrogenase (513 mU mg^−1^) respectively. In addition to ethanol, glycerol fermentation by *A. acetethylicum* also produced small amounts of acetate and formate. However, no activity could be measured for the phosphate‐dependent conversion of acetyl‐CoA to acetyl‐phosphate by phosphate acetyltransferase, but a comparatively lower activity of 26 mU mg^−1^ was observed for an ADP‐dependent acetate kinase in cell‐free extract which converts acetyl‐phosphate to acetate and generates ATP. Similar to acetate kinase, a very low activity (1.6 mU mg^−1^) was observed for formate dehydrogenase (Table 3) and a comparably very high activity of 3070 mU mg^−1^ was observed for hydrogenase (BV).

According to the proposed pathway (Fig. 4), fermentation of glycerol needs reoxidation of NADH and ferredoxin. Reoxidation of ferredoxin occurs mainly through hydrogenase, but also by formate dehydrogenase to a lower extent (Table 3). When glycerol, NADH and vitamin B_12_ were combined in buffer along with cell‐free extract of glycerol‐grown cells, no activity was detectable for the assumed glycerol dehydratase and the combined 1,3‐propanediol dehydrogenase, indicating that glycerol is unlikely to serve as an electron acceptor to produce 1,3‐propanediol via 3‐hydroxypropionaldehyde. Furthermore, no activity for glycerol dehydrogenase was observed in the cell‐free extract of glucose‐grown cells, indicating that the latter enzyme is specifically expressed during growth with glycerol.

## Discussion

Glycerol was studied as a substrate for biofuel production mainly because of its abundance, low price and its highly reduced state that makes it prone to generate reduced products like ethanol, hydrogen and also other industrially relevant compounds (Dharmadi *et al*., 2006; Clomburg and Gonzalez, 2013). In comparison with other glycerol‐fermenting strains, *A. acetethylicum* has higher or at least similar glycerol tolerance, but a low ethanol tolerance. *Clostridium pasteurianum* converts 691 mM (63.6 g l^−1^) of glycerol to mixed fermentation products including butanol, 1,3‐propanediol, ethanol, butyrate, acetate and lactate, when grown with 1250 mM (114.6 g l^−1^) of initial glycerol concentration (Biebl, 2001). Glycerol fermentation by *E. coli* at an initial glycerol concentration of 108 mM (10 g l^−1^) yields mainly ethanol, hydrogen and formate, similar to *A. acetethylicum*, but requires complex growth supplements such as yeast extract, tryptone or corn steep liquor (Dharmadi *et al*., 2006; Murarka *et al*., 2008). Similarly, *Paenibacillus macerans,* a glycerol‐fermenting bacterium, produces ethanol and 1,2‐propanediol but depends as well on tryptone as supplement in the growth medium ((Table 4); Gupta *et al*., 2009). In contrast to these reports, *Anaerobium acetethylicum* did not require additional organic supplements for fermentation of glycerol, except for the defined seven vitamins (Pfennig, 1978) present in the medium which include biotin. Biotin could replace yeast extract in cultures of *C. pasteurianum*, but the overall fermentation time was three times longer than with yeast extract (Biebl, 2001). When grown in defined mineral medium containing the seven vitamins and glycerol as substrate, *A. acetethylicum* had growth rates of 0.101–0.116 h^−1^ which are about 2–3 times higher than those reported for *E. coli* (0.04 h^−1^) when grown with glycerol in the presence of tryptone (Murarka *et al*., 2008), but about four times lower than those reported for *P. macerans* ((0.4 h^−1^); Gupta *et al*., 2009). However, growth of *A. acetethylicum* was not exponential any more after 44 h, indicating that growth is inhibited at this time point. Exponential growth in defined medium is basically possible when grown with gluconate at a growth rate of 0.693 h^−1^; therefore, the medium itself should allow exponential growth as well for cells grown with glycerol (Patil *et al*., 2015).

**Table 4:** Comparison of ethanol production and growth rates between *A. acetethylicum* and other anaerobic glycerol‐fermenting bacterial strains

Organisms	Max. glycerol tolerated (M; (g l^−1^))	Organic supplements required	Fermentation products	Max. growth rate observed (h^−1^)	References
*Anaerobium acetethylicum*	1.5 (138)	7‐vitamins	E, H, a,f, pg, CO_2_	0.116	This study
*Escherichia coli* MG1655	0.108 (10)	CTS	E, (H, F)^a^,s, CO_2_	0.04	Dharmadi *et al*. (2006)
*Escherichia coli* SS1	0.375 (34.5)	T, YE	E, (H, F),^a^ s, CO_2_	No data available	Adnan *et al*. (2014)
*Paenibacillius macerans*	0.108 (10)	T	E, H, pg, f	0.4	Gupta *et al*. (2009)
*Clostridium pasteurianum*	1.25 (115)	Biotin	E, 1,3‐Pd, ButOH, B, A	0.37	Biebl (2001)

Fermentation products H = hydrogen, E = ethanol, F/f = formate, S/s = succinate, Pg/pg = propylene glycol, ButOH = butanol, 1,3‐Pd = 1,3‐propanediol, B = butyrate, A/a = acetate (capital letters – major products and small letter – minor products); complex supplements: CTS = corn steep liquor, T = tryptone, YE = yeast extract.

**a.** Production of hydrogen or formate is pH dependent.

In addition, the maximum glycerol concentration tolerable by *A. acetethylicum* was 1500 mM (138 g l^−1^), which is 13.8 times higher than the glycerol concentrations tested for *E. coli* (Dharmadi *et al*., 2006; Murarka *et al*., 2008). In a study aimed at optimizing glycerol utilization by *E. coli*, the optimal glycerol concentration was 375 mM (34.5 g l^−1^; Adnan *et al*., 2014). However, even though *A. acetethylicum* can grow at comparably high initial glycerol concentrations, maximally tolerable ethanol concentrations reached during glycerol fermentation were in the range of 60–70 mM, which is similar to the maximal concentrations observed for *E. coli* (Dharmadi *et al*., 2006; Murarka *et al*., 2008), but much lower than the observed maximum ethanol concentration of 342 mM (15.72 g l^−1^) for a growth‐optimized *E. coli* strain (Adnan *et al*., 2014). However, it is unclear whether the ethanol concentrations accumulating in cultures of the latter two bacteria do not increase further due to lysis of the cells by ethanol or due to thermodynamic inhibition. This is, however, unlikely as the overall free reaction enthalpy of glycerol conversion to ethanol and hydrogen is negative enough to allow complete conversion of substrate into product (Eq. (1)).

(1) $$\begin{array}{c} C_{3}H_{8}O_{3}\to C_{2}H_{6}O+H_{2}+{\text{CO}}_{2}\;\Delta G^{0^{\prime }}=-87.6\;\text{kJ}\;{\text{mol}}^{-1}. \end{array}$$

The free reaction enthalpy required to generate one ATP from phosphorylation of ADP to ATP is about −60 to −70 kJ mol^−1^ (Schink, 1997). Therefore, equation (1) should allow the production of at least 1 mole of ATP per mole of glycerol. When considering the reaction of glycerol fermentation carried out by cultures of *A. acetethylicum*, which also produced small amounts of side products, the reaction becomes even more favourable allowing an overall ATP yield of 1–2 ATP per mole of glycerol (Eq. (2)).

(2) $$\begin{array}{cc} & C_{3}H_{8}O_{3}\to 0.82\;C_{2}H_{6}O+1\;H_{2}+1.1\;{\text{CO}}_{2}+0.11\;C_{2}H_{3}O_{2}^{-}\\ & \;\;\;+0.11\;H^{+}+0.03\;C_{3}H_{8}O_{2}\;\;\;\Delta G^{0^{\prime }}=-145.2\;\;\;\;k\;\;J\;{\text{mol}}^{-1} \end{array}$$

when Eq. (1) reaches its equilibrium (ΔG’ = 0), including 60 kJ mol^−1^ for formation of 1 ATP, the equilibrium constant has a value of about 10^5^, meaning that the reaction equilibrium is far on the side of the reaction products. Thus, thermodynamic inhibition can be ruled out as a possible reason for the incomplete fermentation of glycerol to ethanol and hydrogen, and inhibition of growth by fermentation metabolites is very likely, i.e., via solvent toxicity. *A. acetethylicum* could ferment glycerol ranging from 10 to 1500 mM initial concentrations, but ethanol production did not exceed 63 mM (Fig. 3). Therefore, increased concentrations of glycerol in the growth medium did not increase ethanol production beyond this latter concentration and growth was most likely inhibited by the ethanol toxicity. Ethanol is known as a growth‐inhibiting agent for bacteria as it acts as hydrophobic stressor, especially at concentrations higher than 25% (w/v; 5400 mM), and therefore destabilizes biological membranes by weakening hydrophobic interactions (reviewed in Cray *et al*., 2015; Ingram, 1990). This effect on hydrophobic interactions also causes a reduction of water activity, which was shown to induce water stress in fungi (Hallsworth *et al*., 1998). This could explain the higher ethanol concentration observed after 166 h of incubation in late stationary to decline phase (62 mM) compared with the ethanol concentration present after 91 h in early stationary phase (38 mM). Most likely, cells were partially lysed by ethanol after they reached stationary phase while still being metabolically active and continuing to ferment glycerol to ethanol and hydrogen. This lytic effect of ethanol might also be reflected by the fact that growth yields decreased with increasing substrate and ethanol concentrations. Consequently, a certain proportion of assimilated substrate was possibly underestimated, which might explain the incomplete electron recoveries at glycerol concentrations higher than 500 mM. Growth could also be inhibited through acidification of the medium. At a substrate concentration of 100 mM of glycerol, the pH dropped from initial 7.2 to 6.3 at the end of growth and earlier investigations revealed a pH range of 6.5–8.5 of strain GluBS11T for growth with gluconate (Patil *et al*., 2015). Therefore, glycerol fermentation could possibly be improved by increasing the buffer strength.

In this study, the fermentation of glycerol to ethanol, CO_2_ and hydrogen by *A. acetethylicum* was biochemically characterized (Fig. 4). Glycerol fermentation by *A. acetethylicum* also produced small amounts of 1,2‐propanediol (Table 1). Clomburg and Gonzalez (2013) reported that 1,2‐propanediol is derived from dihydroxyacetone phosphate. The enzyme methylglyoxal synthase which dephosphorylates dihydroxyacetone phosphate to methylglyoxal was identified in the proteome of *A. acetethylicum* (Table 3). Methylglyoxal (2‐oxopropanal) could theoretically be reduced to 1,2‐propanediol via acetol (hydroxyacetone) or lactaldehyde (Clomburg and Gonzalez, 2011). Both pathways involve glycerol dehydratase and aldehyde oxidoreductase, of which one at least the gene for glycerol dehydratase could be identified in the genome of *A. acetethylicum* (glycerol dehydratase large subunit Ga0116910_100557). 2‐oxopropanal could therefore possibly be reduced to 1,2‐propanediol by one or both of the aforementioned pathways (Fig. 4). 2‐oxopropanal is also known as a highly toxic metabolite in bacterial cells and could therefore be inhibitory for growth of *A. acetethylicum* (Booth *et al*., 2003; Clomburg and Gonzalez, 2011). Despite the fact that HPLC chromatograms of culture supernatants of *A. acetethylicum* occasionally showed small peaks at the same retention time as 2‐oxopropanal, accumulation of this metabolite could not be reliably verified and 2‐oxopropanal did possibly not exceed concentrations higher than 1 mM (data not shown).

Interestingly, batch fermentation experiments with glycerol revealed that hydrogen accumulated at high concentrations, while almost no formate was produced (Table 1). This finding was supported by the approximately 2000‐fold lower activity of formate dehydrogenase compared with hydrogenase (Table 3). In contrast to this, cultures grown with gluconate or glucose as substrates produced higher concentrations of formate than hydrogen at a ratio of hydrogen to formate of about 1:2 (Patil *et al*., 2015). Therefore, formate is a more prominent metabolite when cultures are grown with sugars, which might be due to the fact that twice as much CO_2_ is released per mole of hexose oxidized compared with glycerol. Even though formate could be detected as a metabolite and formate:benzyl viologen oxidoreductase activity was detected in cell‐free extracts, we were unable to find the corresponding formate dehydrogenase genes in the genome sequence. Possibly, the observed activity is a side reaction of formate C‐acetyltransferase (pyruvate:formate lyase) which was identified in the proteome.

When *A. acetethylicum* was grown with a larger headspace‐to‐culture volume ratio, the maximum concentrations of ethanol and hydrogen were slightly higher compared with cultures with a low headspace‐to‐culture volume ratio, indicating that hydrogen is also inhibitory for glycerol degradation. Similar observations were also made for *E. coli* grown in a fermenter sparged with argon gas, which vastly increased the amount of glycerol degraded (Murarka *et al*., 2008). Although experiments with cultures of *A. acetethylicum* permanently sparged with argon or nitrogen have not been done yet, it can be assumed that this might stimulate fermentation of glycerol as well.

Even though *A. acetethylicum* has certain advantages over other glycerol‐fermenting organisms, the fact that side products are formed in batch cultures especially at higher glycerol concentrations might be disadvantageous for large‐scale bioethanol production. Among acetate, formate and 1,2‐propanediol frequently observed at small concentrations, a further product was released in cultures of *A. acetethylicum* that could not be identified by HPLC. It was previously reported that during fermentation of glycerol by *Clostridium pasteurianum*, 1,3‐propanediol is produced via 3‐hydroxypropionaldehyde (Dabrock *et al*., 1992). However, in this study we could detect neither 1,3‐propanediol nor 3‐hydroxypropionaldehyde as metabolites, and the activities of the respective enzymes were absent in *in vitro* assays, although we recently reported that occasionally very small amounts of 1,3‐propanediol could be detected by HPLC in cultures of *A. acetethylicum* (Patil *et al*., 2015). Likewise, succinate, lactate, 1‐butanol, 1‐propanol, 2‐propanol, butyrate, propionate and 1,3‐butanediol were ruled out as possible side products and neither one of the corresponding metabolic pathways is present in the genome of *A. acetethylicum* with a complete set of genes (Patil *et al*., 2016). Interestingly, the percentage of ethanol produced per glycerol slightly increased with increasing substrate concentration, with an optimal initial glycerol concentration of 100 mM (9.2 g l^−1^) at which the strain showed maximal efficiency of glycerol‐to‐ethanol conversion (79% glycerol conversion to ethanol).

## Conclusion

As *A. acetethylicum* strain GluBS11^T^ naturally has a high tolerance towards elevated glycerol concentrations, it could be a potentially useful agent for treating glycerol‐rich wastewaters coming from the biodiesel industries. Our study shows that the maximum initial glycerol concentration that did not inhibit growth and metabolic activity of the cells was 1500 mM of pure glycerol. However, 100 mM of initial glycerol concentration was optimal for efficient conversion of glycerol to ethanol (79% conversion efficiency) and hydrogen as compared to other tested initial concentration. Solvent toxicity tests of *A. acetethylicum* for glycerol and ethanol showed that ethanol is the key solvent that strongly inhibits growth and fermentation activity. Although the strain could produce about 61 mM of ethanol during growth with 100 mM of glycerol, addition of 50 mM of initial ethanol completely inhibited growth (Fig. S1). The inability to accumulate high concentrations of ethanol (more than 60 mM) during growth is possibly a drawback for large‐scale applications. Moreover, pure glycerol was used as a substrate in this study and glycerol derived from biodiesel production might contain compounds that inhibit growth. Yet, due to its high tolerance for glycerol and its fermentation pattern to mainly ethanol and hydrogen, the strain has a high potential for future industrial application in biodiesel industries to convert crude glycerol to value‐added biofuel. Future research should therefore focus on increasing the strain's ability to tolerate ethanol concentrations higher than 60 mM. This could be accomplished, e.g. by genetic engineering of the strain and introducing metabolic pathways for synthesis of oleic acid, which is believed to protect the cell membranes of yeast from the toxic effect of ethanol (You *et al*., 2003). This would, however, require the development of a genetic system for the strain. It is known that ethanol tolerance in yeast can also be increased by addition of Tween‐80 and oleic acid to growth media, which did not increase ethanol tolerance in *A. acetethylicum*, however, (data not presented) and probably led to inhibition of growth (Andreasen and Stier, 1954). Besides ethanol and hydrogen as main fermentation products, the strain produces very little amount of undesired fermentation products such as acetate and formate; therefore, future efforts in metabolically engineering the strain could aim at deleting the enzymes leading to acetate production, i.e., acetate kinase, and formate production, i.e., formate dehydrogenase, for the production of bioethanol.

## Experimental procedures

### Source of strain and genome sequence

*Anaerobium acetethylicum* strain GluBS11^T^ was recently isolated in our laboratory and was characterized morphologically and taxonomically (Patil *et al*., 2015). Strain GluBS11^T^ is available in public culture collections such as the German Collection of Microorganisms and Cell Cultures (DSM 29698) or the Korean Type Culture Collection (KCTC 15450). Recently, metabolic features and characteristics of the high‐quality permanent draft genome sequence of *A. acetethylicum* strain GluBS11^T^ are described (Patil *et al*., 2016). The draft genome of *A. acetethylicum* strain GluBS11^T^ was sequenced as part of the Genomic Encyclopedia of Type Strains, Phase III (KMG‐III): the genomes of soil and plant‐associated and newly described type strains (Whitman *et al*., 2015). The genome project is deposited in the Genomes OnLine Database under Project ID: Gp0139288 (Liolios *et al*., 2008).

### Cultivations and glycerol fermentation experiment

Precultures of *A. acetethylicum* were cultured in a bicarbonate‐buffered and sulfide‐reduced mineral medium adjusted to pH 7.2 as described before (Patil *et al*., 2015) containing 10 mM of glycerol, at 30°C in 25 ml Hungate test tubes or 100 ml infusion bottles. The following seven vitamins were added to the medium after autoclaving from a concentrated, filter‐sterilized stock solution: cyanocobalamine (50 μg l^−1^), p‐aminobenzoic acid (50 μg l^−1^), biotin (10 μg l^−1^), nicotinic acid (100 μg l^−1^), pantothenate (25 μg l^−1^), pyridoxamine (250 μg l^−1^) and thiamine (50 μg l^−1^; Patil *et al*., 2015; Pfennig, 1978). For longer storage, the strain was maintained in culture medium at 4°C, and actively growing cultures were used as inoculum for inoculation of each experiment. Fermentation experiments were performed either in 25 ml glass tubes sealed with butyl rubber stoppers and closed with aluminium crimps under a N_2_/CO_2_ (80:20) atmosphere filled with 10 ml of medium, or in infusion bottles sealed with butyl rubber stoppers under the same atmosphere filled with each of 50, 100 ml or 1 l of culture medium. Growth experiments with large headspace‐to‐culture volume ratio were performed in 600 ml infusion bottles sealed with butyl rubber stoppers. The gas phase of the bottles was exchanged to N_2_/CO_2_ (80:20). Then, the bottles were autoclaved at 121°C and 1 bar overpressure for 25 min and thereafter filled with 20 ml of medium with syringes. Substrate stock solutions (glycerol and ethanol) were maintained under anoxic conditions (under N_2_ gas) and filter‐sterilized using cellulose acetate filters with 0.2 μm pore size. Stock solutions were added to the medium as sole source of carbon and energy with sterile disposable needles and syringes. To investigate the influence of different concentrations of glycerol on the fermentation pattern, glycerol was added to the culture medium at concentrations ranging from 10 to 200 mM. Growth was monitored by measuring optical densities directly in test tubes at 600 nm wavelength (OD600 nm) using a tube spectrophotometer M107 (Spectronic Camspec, Leeds, UK). When cultures reached optical densities higher than 0.7, samples were withdrawn with syringes and diluted 1:10 with medium and optical densities were measured in plastic cuvettes with a Jenway 6305 spectrophotometer (Jenway, Staffordshire, UK). Spectrophotometers were zeroed with sterile blank medium, and all experiments were performed in triplicates.

### Alcohol (Glycerol and Ethanol) tolerance

To investigate the influence of glycerol and ethanol at different concentrations on glycerol fermentation by *A. acetethylicum*, various concentrations of glycerol (ranging from 10 mM to 3000 mM) and ethanol (10–100 mM) were added to the culture medium. *A. acetethylicum* cells were always grown and maintained in glycerol‐containing medium and gradually adapted to higher concentrations of glycerol. Cultures were inoculated to an initial OD600 of about 0.02 in medium supplemented with different concentrations of glycerol. In the ethanol tolerance experiments, all culture tubes were supplemented with 10 mM of glycerol as growth substrate with varying concentrations of ethanol. All incubations were performed at 30°C in the dark, and growth was monitored spectrophotometrically at OD600 until the cultures reached the stationary phase. At the end of experiments (stationary phase), samples were collected from each culture tube and stored at −20°C until further use. Measurement of substrate consumption or fermentation product formation was taken by HPLC as described below. All fermentation experiments were performed in triplicates.

### Preparation of cell‐free extracts

Cultures of *A. acetethylicum* grown in 1 l medium with 20 mM of glycerol were grown until cells reached mid‐to‐late exponential growth phase (after 24–48 h). Cells were harvested by centrifugation at 7000 × g, for 20 min at 10°C in a Sorvall RC‐5B centrifuge (Du Pont de Nemours, Bad Homburg, Germany) under anoxic conditions using airtight polypropylene centrifuge bottles in an anaerobic chamber (Coy, Ann Arbor, MI, USA). Cell pellets were washed by resuspending them in approximately 200 ml of anoxic 50 mM of Tris–HCl buffer (pH 7.5) containing 3 mM of dithiothreitol (DTT) and centrifuged again. Cell pellets were stored at −20°C until further use. Finally, cell pellets were suspended in 4–5 ml of the same buffer and cells were disrupted by passing three times through an ice‐cold MiniCell French pressure cell (SLM Aminco, Cat. No. FA003) operated at 137 MPa pressure as described recently (Junghare *et al*., 2016) or cells were opened by enzymatic lysis using lysozyme treatment. Lysis with lysozyme was performed by adding 2 mg ml^−1^ of lysozyme and 0.1 mg ml^−1^ of DNase I to cell suspensions, which were then incubated at 37°C for 1 h. Cell debris was removed by high‐speed centrifugation (27 000 × g for 30 min at 4°C) using an ultracentrifuge under anoxic conditions. The supernatant was transferred to 8 ml serum vials closed with butyl rubber stoppers and sealed with aluminium caps. The headspace was exchanged under a stream of 100% nitrogen gas. This supernatant was defined as crude cell‐free extract and was stored on ice for enzyme activity measurements.

### Total proteome analysis

Cell‐free extracts of glycerol‐grown cells of *A. acetethylicum* were used for the identification of the total proteome to identify the putative proteins/enzyme involved in the anaerobic fermentation of glycerol. Proteins were reduced with 10 mM of DTT for 30 min and alkylated with iodoacetamide followed by overnight trypsin digestion. The resulting digested protein mixture was applied to reversed‐phase liquid chromatography nanospray tandem mass spectrometry (LC‐MS/MS) using an LTQ‐Orbitrap mass spectrometer (Thermo Fisher) and an Eksigent nano‐HPLC. The LC‐MS/MS was equipped with the reversed‐phase LC column measuring 5 μm, 100 Å pore size C18 resin in a 75‐μm i.d. × 10 cm long piece of fused silica capillary (Acclaim PepMap100; Thermo Scientific, Dreieich, Germany). After sample injection, the column was washed for 5 min with 95% mobile phase A (0.1% formic acid) and 5% mobile phase B (0.1% formic acid in acetonitrile), and peptides were eluted using a linear gradient of 5% mobile phase B to 50% mobile phase B in 205 min, then to 80% B in an additional 5 min at 300 nl min^−1^. The LTQ‐Orbitrap mass spectrometer was operated in a data‐dependent mode in which each full MS scan (30 000 resolving power) was followed by seven MS/MS scans where the seven most abundant molecular ions were dynamically selected and fragmented by collision‐induced dissociation (CID) using a normalized collision energy of 35% in the LTQ ion trap. Dynamic exclusion was allowed. Tandem mass spectra were searched against a suitable protein database of the annotated genome sequence of *A. acetethylicum* using Mascot (Matrix Science) with trypsin enzyme cleavage, static cysteine alkylation by iodoacetamide and variable methionine oxidation. Search results were validated on the basis of top hits and scores obtained in the Mascot search engine.

### *In vitro* enzyme activity measurements

For measurement of *in vitro* enzyme activities, cell‐free extract was prepared from cells grown with glycerol or glucose (the latter as control) as described above and used for enzyme assays. Enzyme activities were measured under anoxic conditions using 1.5 ml quartz cuvettes closed with rubber stoppers and gassed with N_2_ gas (unless otherwise mentioned). All additions were performed with airtight Unimetrics microlitre syringes (Macherey‐Nagel, Germany). Enzyme assays were performed at 30°C using a UV–vis spectrophotometer V‐630 (Jasco, Gross‐Umstadt, Germany). One unit of specific enzyme activity (U) was defined as the amount of enzyme required to convert 1 μmole of substrate into the specific product per minute and per milligram of protein. Protein concentrations were estimated by the microprotein assay (Bradford, 1976) with bovine serum albumin as standard. All enzyme assays were performed at least in triplicates under anoxic conditions at 30°C.

- **Glycerol dehydrogenase** (glycerol:NAD^+^ oxidoreductase, EC 1.1.1.6) activity was assayed using 1.5 ml in quartz cuvettes containing 1 ml of reaction mixture containing 50 mM of Tris–HCl buffer, pH 7.5, 3 mM of dithiothreitol, 0.2 mM of NAD^+^ and 20 μl of cell‐free extract (approx. 0.1 mg of protein). Enzyme reactions were started by addition of 20 mM of glycerol. The reduction of NAD^+^ to NADH in the presence of glycerol was monitored spectrophotometrically by the increase in absorbance due to NADH formation at 340 nm wavelength.
- **Glycerol dehydratase** (EC 4.2.1.30) **and 1, 3‐propanediol dehydrogenase** (EC 1.1.1.202) activities were assayed together in a coupled enzyme assay containing 1 ml of reaction mixture of 50 mM of Tris–HCl, pH 7.6, with 3 mM of DTT, 0.3 mM of NADH, 24 μM of cyanocobalamine (vitamin B_12_) and 20 μl of cell‐free extract. The reaction was started by addition of 2 mM of glycerol and decrease in the NADH concentration was determined at 340 nm in spectrophotometer.
- **Glyceraldehyde‐3‐phosphate dehydrogenase** (EC 1.2.1.12) was measured following the increase in NADH concentration at 340 nm. The 1 ml of reaction mixture containing 50 mM of potassium phosphate buffer, pH 7.5, 0.2 mM of NAD^+^ and 20 μl of cell‐free extract. The reaction was started by addition of 0.3 mM of glyceraldehyde 3‐phosphate.
- **Pyruvate synthase** (EC 1.2.7.1; also called pyruvate:ferredoxin oxidoreductase) was assayed as reduction of benzyl viologen monitored at 578 nm. The 1 ml of assay mixture containing 50 mM of Tris–HCl buffer, pH 7.5, containing 3 mM of DTT, 2 mM of benzyl viologen, 0.3 mM of CoASH, and 20 μl of cell‐free extract. The reaction was started by addition of 2 mM of pyruvate. Alternative to benzyl viologen, the enzyme activity was also determined with ferredoxin isolated from *Clostridium pasteurianum*. The reduction of ferredoxin was monitored spectrophotometrically as the increase in absorbance at 390 nm wavelength. Ferredoxin from *C. pasteurianum* was essentially prepared as previously described (Schönheit *et al*., 1978).
- **Acetate kinase** (EC 2.7.2.1) was measured in a coupled enzyme assay. One millilitre of reaction mixture in 50 mM of Tris–HCl, pH 7.6, with 3 mM of DTT, 0.33 mM of NADH, 5 mM of phosphoenolpyruvate, 5 mM of MgCl_2_, 5 mM of ATP, 2 U of D‐lactate dehydrogenase and 20 μl of cell‐free extract. The reaction was started with 5 mM of acetate, and decrease in concentration of NADH was monitored at 340 nm.
- **Acetaldehyde dehydrogenase** (EC 1.2.1.10) activity was assayed in 1 ml of reaction mixture of 50 mM of Tris–HCl buffer, pH 7.5, containing 3 mM of DTT, 0.2 mM of NAD^+^, 0.3 mM of coenzyme A (CoASH) and 20 μl of cell‐free extract. The reaction was started by addition of 0.5 mM of acetaldehyde. The reduction of NAD^+^ to NADH was monitored spectrophotometrically by the increase in absorbance at 340 nm wavelength.
- **Alcohol dehydrogenases** (EC 1.1.1.1) activity was determined in 1 ml of reaction mixture containing 50 mM of Tris–HCl buffer, pH 7.5, 3 mM of dithiothreitol, 0.2 mM of NADH and 20 μl of cell‐free extract. The enzyme reaction was started by addition of 0.5 mM of acetaldehyde, and the decrease in NADH absorbance was monitored spectrophotometrically at 340 nm wavelength.
- **Formate dehydrogenase** (EC 1.1.99.33) activity was assayed as reduction of benzyl viologen monitored at 578 nm. The 1 ml of assay mixture contained 50 mM of Tris–HCl buffer, pH 7.5, 3 mM of DTT, 2 mM of benzyl viologen and 20 μl of cell‐free extract. The reaction was started by addition of 2 mM of formate.
- **Hydrogenase** (EC 1.12.99.6) activity was assayed in cuvettes gassed with hydrogen. The 1 ml of assay mixture containing 50 mM of Tris–HCl buffer, pH 7.5, containing 3 mM of DTT, 2 mM of benzyl viologen. The reaction was started by addition of 20 μl of cell extract, and reduction of benzyl viologen was monitored at 578 nm.

### Analytical methods

Ethanol, acetate, glycerol, propionate and formate were quantified by HPLC with a Rezex RHM‐monosaccharide H^+^ 300 × 7.80 mm 8 μm ion exchange column (Phenomenex, Aschaffenburg, Germany) using a method previously described (Dharmadi *et al*., 2006). The column was operated at 40°C and 30 mM of sulfuric acid was used as mobile phase at a flow rate of 0.6 ml min^−1^ using a LC‐10ATvp pump (Shimadzu, Munich, Germany). The mobile phase was degassed with a DGU‐20A3R degassing unit (Shimadzu), and samples were injected with a 234 autosampler (Gilson, Limburg‐Offheim, Germany). For analysis of the separated compounds, a refractive index detector RID‐10A (Shimadzu) was used and the signals obtained were analysed with the shimadzu lab solutions software version 5.81. When 5 mM of sulfuric acid was used as eluent with the column heated to 60°C, formate and glycerol could not be separated. Similar observations were made for the same separation problem before (Dharmadi and Gonzalez, 2005). Prior to HPLC analysis, samples and standards were prepared by mixing 200 μl of sample or standard with 20 μl of 1 M H_2_SO_4_ to remove ${\text{HCO}}_{3}^{-}$. Acrolein as a standard was measured with and without prior acidification. 3‐hydroxypropionaldehyde is not commercially available and was prepared by acidification of acrolein (2‐propenal) with sulfuric acid essentially as described before (Hall and Stern, 1950). Acrolein was added to an anoxic 1 M H_2_SO_4_ solution to a final concentration of 100 mM. This solution was incubated at room temperature overnight (16 h) and directly used as a standard for HPLC analysis.

Hydrogen was quantified with a GC6000 Vega Series 2 GC (Carlo Erba, Italy) gas chromatograph equipped with a HWD 430 thermal conductivity detector. The steel column (45/60 Carboxen 1000, Supelco) was heated to 120°C. A comparable column was used as reference column. Nitrogen was used as carrier gas at a concentration of 100% and at a column pressure of 60 kPa. The manual injection port was heated to 150°C, and the detector and filament temperatures of the thermal conductivity detector were 180°C and 270°C respectively. Detector signals were analysed with a D‐2500 Chromato‐Integrator (Merck Hitachi) set to a plot attenuation of 4 and a sensitivity of 250. Samples of 100 μl were withdrawn from culture vessels with a syringe mounted to a valve with needle and injected at atmospheric pressure.

### Thermodynamic calculations

The assimilation equation for glycerol fermentation was calculated assuming that glycerol is oxidized to biomass with CO_2_. Thermodynamic calculations were done according to method described (Thauer *et al*., 1977) and using the online ΔG calculator tool available on our webpage ( https://cms.uni-konstanz.de/schink/dg-calculator/). The free energy formation enthalpy for 1,2‐propanediol is ΔH_f_^0^_liquid_ propylene glycol = 501.0 ± 4.1 kJ mol^−1^ (Knauth and Sabbah, 1990), which was not available in the list mentioned in Thauer *et al*., (1977) and was retrieved from the webpage of the National Institute of Standards and Technology webbook ( http://webbook.nist.gov/cgi/inchi?ID=C4254142&Units=SI).

### Source of chemicals

All biochemicals used in this study were purchased from Sigma‐Aldrich (Munich, Germany) or Carl Roth (Karlsruhe, Germany) and were at least of reagent grade. All other chemicals were from Sigma‐Aldrich and were of analytical grade. Acrolein was a kind gift from Christian Leitner from the group of Dr. Tanja Gaich at the University of Konstanz. Gases were obtained from Messer‐Griesheim (Darmstadt, Germany) or Sauerstoffwerke Friedrichshafen (Friedrichshafen, Germany).

## Author's contribution

YP and MJ contributed equally. Experiments were conducted by YP, MJ and NM. NM, YP and MJ designed experiments and wrote the manuscript.

## Conflict of interest

The authors declare no conflict of interest.

## Supporting information


**Fig. S1.** Growth of *A. acetethylicum* at different initial concentrations of glycerol and ethanol. Shown are mean values of triplicates ± standard deviations.
