# Supplementary figures and images for: Fermentation of glycerol by Anaerobium acetethylicum and its potential use in biofuel production
Source: Microb Biotechnol. 2016 Dec 22;10(1):203–17. doi: 10.1111/1751-7915.12484 (PMC5270724; doi:10.1111/1751-7915.12484)

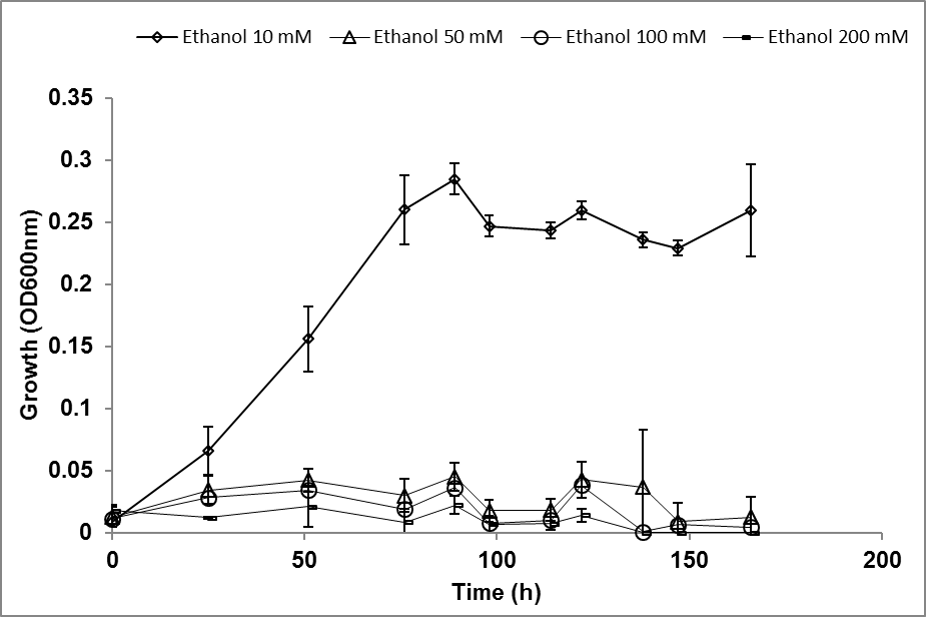

Supplement: Supplementary file 1 — Fig. S1. Growth of A. acetethylicum at different initial concentrations of glycerol and ethanol. Shown are mean values of triplicates ± standard deviations. [file MBT2-10-203-s001.tif]
